# dUTPase modulates mycobacterial homologous recombination and interacts with the AdnAB helicase–nuclease

**DOI:** 10.1093/nar/gkag660

**Published:** 2026-06-27

**Authors:** Rita Hirmondó, Dániel Molnár, Gergely Döbrőssy, Szonja T Kovács, Beáta G Vértessy, Judit Tóth

**Affiliations:** Institute of Molecular Life Sciences, HUN-REN Research Centre for Natural Sciences, Budapest 1117, Hungary; Institute of Molecular Life Sciences, HUN-REN Research Centre for Natural Sciences, Budapest 1117, Hungary; Institute of Molecular Life Sciences, HUN-REN Research Centre for Natural Sciences, Budapest 1117, Hungary; Molecular Medicine Division, Semmelweis University Doctoral College, Budapest 1085, Hungary; Institute of Molecular Life Sciences, HUN-REN Research Centre for Natural Sciences, Budapest 1117, Hungary; Institute of Molecular Life Sciences, HUN-REN Research Centre for Natural Sciences, Budapest 1117, Hungary; Department of Applied Biotechnology and Food Sciences, Faculty of Chemical Technology and Biotechnology, BME Budapest University of Technology and Economics, 1111 Műegyetem rkp. 3, Budapest, Hungary; Institute of Molecular Life Sciences, HUN-REN Research Centre for Natural Sciences, Budapest 1117, Hungary

## Abstract

This study identifies a previously unrecognized interaction between *Mycobacterium tuberculosis* dUTPase (Dut) and the AdnAB homologous recombination complex. Using a combination of yeast two-hybrid screening, mycobacterial protein fragment complementation, and biochemical analyses with purified proteins, we show that dUTPase physically interacts with the N-terminal region of AdnA and modulates the activity of the AdnAB helicase–nuclease complex. Biochemical assays demonstrate that Dut enhances AdnAB activity on DNA substrates and alters the AdnAB–DNA interaction. Mutational perturbation of Dut, including catalytic inactivation or deletion of a mycobacteria-specific surface loop, reduces its stimulatory effect on AdnAB *in vitro* and decreases recombination efficiency in mycobacterial cells. Together, these results support a functional connection between dUTPase and the AdnAB DNA-processing machinery and suggest a potential link between nucleotide metabolism and DNA repair pathways.

## Graphical Abstract

**Graphical Abstract:**
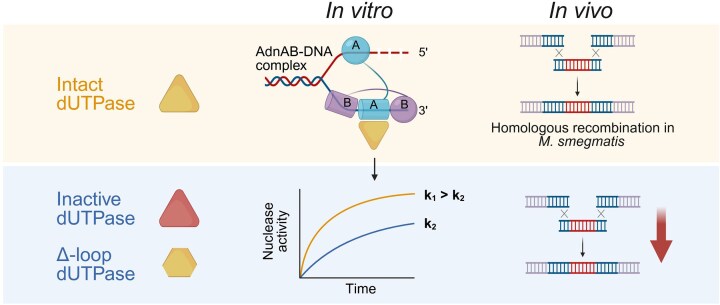

## Introduction

**Figure 1.**
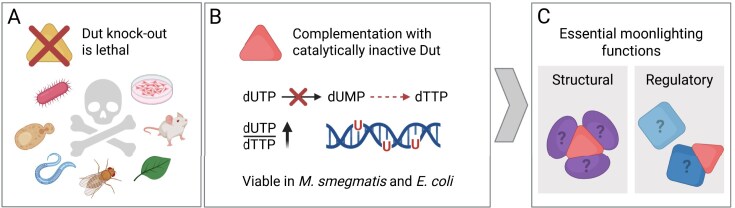
Consequences of dUTPase protein loss versus catalytic inactivation. (**A**) Complete knockout of the Dut protein is lethal in all investigated organisms. (**B**) In *M. smegmatis*, cells remain viable when expressing a catalytically inactive Dut variant, despite elevated genomic uracil levels. (**C**) These findings indicate that, beyond its enzymatic function, Dut likely fulfills additional non-enzymatic structural and/or regulatory roles. Created in BioRender. Toth, J. (2026) https://BioRender.com/u9jsl15.

dUTPases (Duts) are ubiquitous and essential nucleotide pool sanitizing enzymes that help maintain DNA integrity by breaking down dUTP to prevent its accumulation in the dNTP pool, which would otherwise lead to uracil incorporation into DNA [1]. dUTP breakdown also serves dTTP biosynthesis through the production of the obligate dUMP precursor (except for a number of bacterial and archaeal species [2]). Duts were thought to fulfill only these straightforward metabolic roles. However, the Dut enzymatic function alone could not explain several unexpected observations associated with Dut deficiencies induced *in vitro* or identified *in vivo*. In organisms where Dut is encoded in the genome, the knockout (KO) of the gene results in lethality in all investigated organisms, including *Mycobacterium tuberculosis* (*M. tuberculosis*) and *Mycobacterium smegmatis* (*M. smegmatis*) (Fig. 1A) [3–9]. Artificially induced Dut point mutations or depletion by RNA interference (RNAi) often lead to growth defects [10–31], thymidine auxotrophy [5, 13, 16, 32, 33], and various genomic instabilities, including increased uracil incorporation into DNA [14, 34, 35], DNA fragmentation [11, 13, 14, 15, 17, 19, 23, 29, 36], and elevated mutation rates [12, 14, 26, 34, 35].

We previously demonstrated that abolishing the enzymatic activity of Dut does not compromise viability, as the intact protein itself is both necessary and sufficient to sustain the growth of *M. smegmatis*, the avirulent model organism for *M. tuberculosis*, the causative agent of tuberculosis [34]. Similar findings have been reported in *Escherichia coli* (*E. coli*) [4] and bacteriophage T5 [31]. Although loss of Dut activity produced a characteristic mutator phenotype manifested by increased mutation rates, elevated genomic uracil levels, and expansion of the intracellular dUTP pool (Fig. 1B), these changes did not measurably affect the growth rate of *M. smegmatis*, at least under short-term laboratory conditions [34]. Together, these findings point to additional functional role(s) for Dut beyond its established enzymatic activity (Fig. 1C).

In 2010, a new “moonlighting” role for staphylococcal helper phage Duts was proposed, highlighting their involvement in gene expression regulation [30]. Subsequent studies by our lab and others have extensively investigated this regulatory mechanism of Dut from several staphylococcal bacteriophages (ϕ11, 80α, ϕNM1) [37–43] [9, 44]. These studies revealed that helper phage Duts interact, in a dUTP-dependent manner, with the Stl repressor of staphylococcal pathogenicity islands (SaPI) [37]. This interaction releases the repressor, activating virulence genes and increasing the pathogenicity of *Staphylococcus aureus* (*S. aureus*). Additionally, growing evidence indicates that Duts produced by various viruses, including important human pathogens, may play unexpected and novel roles in regulating the host’s innate immune response (for a comprehensive review, see [45]).

**Figure 2.**
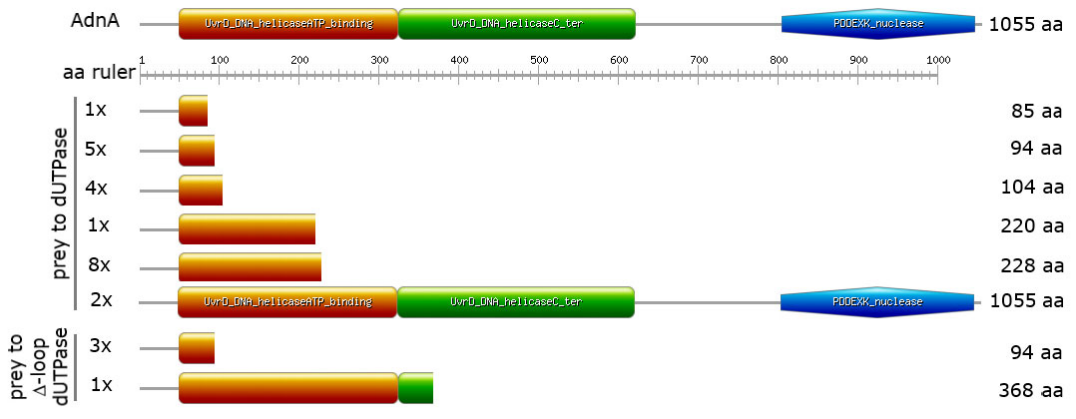
Yeast two-hybrid hits mapped onto the AdnA protein domain structure. The domain structure of *M. tuberculosis* AdnA (gene ID: Rv3202c) was analyzed using the InterProScan tool (https://www.ebi.ac.uk/interpro/result/InterProScan/#table). The UvrD-like DNA helicase ATP-binding domain (profile PS51198) is represented in orange, the UvrD-like DNA helicase C-terminal domain (profile PS51217) in green, and the PD-(D/E)XK nuclease superfamily domain (Pfam PF12705) in blue. “aa” refers to amino acids. The frequencies of prey clones identified are shown on the left of each fragment, while the fragment length is indicated on the right.

Given our findings that mycobacterial Dut has roles beyond its enzymatic activity and our involvement in studying Dut as a gene expression regulator, we sought potential interaction partners for mycobacterial Dut. A yeast two-hybrid screen identified AdnA—a helicase-nuclease initiating HR—as a key interaction partner for *M. tuberculosis* Dut (see below in details in the Results section). Mycobacteria possess highly evolved and redundant DNA repair systems, including base excision repair, nucleotide excision repair, non-canonical mismatch repair, and multiple mechanisms for double-strand break repair (DSBR) [46]. Of all damage, DSBs are the most dangerous ones to dividing cells if unrepaired. While *E. coli* relies solely on HR for DSB repair, mycobacteria utilize three distinct pathways: HR, non-homologous end joining (NHEJ), and single-strand annealing (SSA) [47]. HR is the most accurate DSB repair mechanism and is crucial in dividing cells, where a second chromosome copy is available [47–49]. In mycobacteria, HR is initiated by the AdnAB complex, a heterodimer in which each subunit contains an N-terminal UvrD-like superfamily I helicase motor domain and a C-terminal RecB-like nuclease domain (Fig. 2). Systematic studies by the Shuman and Glickman labs have elucidated how the AdnAB complex generates the resected single-stranded DNA required for strand invasion in HR [50–55]. However, the mechanisms regulating AdnAB activity remain largely unexplored. In particular, it is unclear how stages analogous to Chi-sequence recognition in RecBCD systems are managed. Without knowledge of potential interaction partners, the full scope of AdnAB regulation cannot be defined. Investigating these partners and their interactions remains a practical direction for future studies.

Here we show that the mycobacterial Dut interacts with the AdnA helicase domain and increases the nuclease activity of AdnAB. Furthermore, we found that Dut-deficient *M. smegmatis* cells exhibit reduced recombination efficiency. This interaction suggests a potential regulatory role for Dut in DSBR, broadening our understanding of Dut functions beyond nucleotide metabolism.

## Materials and methods

### Bacterium strains and media

For cloning, *E. coli* XL1-Blue (Novagene, Merck) strain cultured in standard LB medium was used. For culturing *M. smegmatis*, standard Lemco broth [5 g/l Lemco (OXOID), 5 g/l NaCl (Sigma), 10 g/l pepton (Bacto), and 0.5 g/l Tween-80 (Sigma)] or Lemco agar [6.25 g/l Lemco (OXOID), 6.25 g/l NaCl (Sigma), 12.5 g/l pepton (Bacto), and 18.75 g/l agar (Bacto)] was used. In the Mycobacterial Protein Fragment Complementation (M-PFC) assays, *M. smegmatis* was grown using a minimal media, 7H9 broth (Middlebook 7H9 broth, 10% glycerol, bovine albumin fraction V 5 g/l, dextrose 2.00 g/l, catalase 0.004 g/l, oleic acid 0.05 g/l, and sodium chloride 0.85 g/l), and 7H10 agar [Middlebrook 7H10 agar, bovine albumin fraction V 5 g/l, dextrose 2.00 g/l, catalase 0.004 g/l, oleic acid 0.05 g/l, and sodium chloride 0.85 g/l]. Antibiotics were used in the following concentrations, carbenicillin (Car) at 50 μg/ml, kanamycin (Kan) at 20 μg/ml, gentamycin (Gen) at 20 μg/ml, hygromycin B (Hyg) at 100 μg/ml, trimethoprim at 6 μg/ml, and streptomycin (Strep) at 20 μg/ml.

### Plasmid construction for protein expression, M-PFC, and recombination assays

The codon-optimized *M. tuberculosis* AdnA gene, including an N-term histidine tag, a Flag tag, and an enterokinase site, was synthesized and cloned into a pet19b vector using NdeI and BamHI restriction sites, while codon-optimized AdnB was synthesised and cloned into a pet28a vector using NcoI and BamHI restriction sites by Synbio Technologies. A nuclease-dead AdnAB heterodimer harboring an inactivating D935A or D935N mutation in the AdnA nuclease domain and an inactivating D1014A mutation in the AdnB nuclease domain was generated by site-directed mutagenesis. The D935N AdnA-D1014A AdnB variant was used in subsequent experiments as a nuclease-dead (nd) variant.

The pUAB series [56] was used to generate the specific plasmids for M-PFC assay. Primers designed to amplify AdnA, AdnA motor domain and Dut are shown in Supplementary Table S1. Specific sequences were amplified by PCR from pET19b-AdnA (Table 1) and pET19b-Dut [34] using Q5^®^ High-Fidelity DNA Polymerase. The PCR product was purified using the Macherey-Nagel NucleoSpin PCR Clean-Up Kit. Plasmid and insert DNA were digested overnight at 37°C using the restriction enzymes (NEB) listed in Table 1. Plasmid DNA was treated with Antarctic phosphatase (NEB) for 30 min at 37°C and ligated to the specific insert (Table 1) using T4 ligase (NEB) at 16°C overnight. Ligation mixtures were transformed into XL1 blue strains. Constructs were verified by sequencing.

**Table 1.** Plasmids used in this study

Plasmid	Vector backbone	Restriction site	Insert	Fusion	Antibiotics	Purpose	Ref.
pET19b-dut	pET19b	N/A	Dut	N-term HIS tag	Car	Recomb. protein expression	[34]
pET19b-AdnA	pET19b	NdeI, BamHI	Adna	N-term HIS, Flag tag, enterokinase site	Car		This study
pET28a-AdnB	pET28a	NcoI, BamHI	AdnB	no	Kan		
pUAB100	N/A	N/A	Leu-zip homodim. domain	C-term mDHFR[F1, 2]	Hyg	MPFC assay control plasmids	[56]
pUAB200			empty	C-term mDHFR[F3]	Kan		
pUAB300				N-term mDHFR[F1, 2]	Hyg		
pUAB400				N-term mDHFR[F3]	Kan		
pUAB_AdnA_C	pUAB100	BamHI, ClaI	AdnA	C-term mDHFR[F1, 2]	Hyg	MPFC assay	This study
pUAB_motor_C	pUAB100	BamHI, ClaI	AdnA motor domain	C-term mDHFR[F1, 2]	Hyg		
pUAB_Dut_C	pUAB200	MfeI, ClaI	Dut	C-term mDHFR[F3]	Kan		
pUAB_Dut_N	pUAB400	EcoRI, HindIII	Dut	N-term mDHFR[F3]	Kan		
pLL_trunc_KanR	pLL192	EcoRV	KanR 3′ fragment	N/A	Strep	Recombination assay	This study
pLL_Rec	pLL_trunc_KanR	NotI, AvrII	L5 integrase cassette	N/A	Strep		
		NotI, KpnI	KanR 5′ fragment	N/A			

The pLL192 vector [57] was used to generate the specific plasmid containing StrepR and N- and C-terminal fragments with the same homology region of the KanR gene used for the recombination assay. The primers used for cloning are listed in Supplementary Table S1. First, the entire pLL192 was amplified from Arg42 of the KanR gene and ligated with T4 ligase to obtain the 5′ truncated KanR-encoding pLL192 plasmid. The KanR and L5 integrase cassettes were amplified by PCR from p2NIL [58] and pUC-Gm_Int [59], respectively, using Q5^®^ High-Fidelity DNA Polymerase. Downstream cloning steps were similar to those described above.

### Generation of *M. smegmatis* strains

The constructs were electroporated into wt, Δloop-Dut [3], or inactive-Dut [34]*, M. smegmatis* electrocompetent cells (2500 V, 25 uF, 5 ms in Eppendorf electroporation device) and selected on the appropriate antibiotics. The constructed strains are described in Table 2.

**Table 2.** Mycobacterial strains used in this study

Strain	Genotype	Promoter	Purpose	Resistance
MPFC(+)	pUAB100; pUAB200	hsp60	M-PFC positive control	Kan, Hyg
MPFC(-)	pUAB300; pUAB200	hsp60	M-PFC negative control	Kan, Hyg
MD-DUTC	pUAB_motor_C; pUAB_Dut_C	hsp60	M-PFC assay	Kan, Hyg
MD-DUTN	pUAB_motor_C; pUAB_Dut_N	hsp60		Kan, Hyg
FL-DUTC	pUAB_AdnA_C; pUAB_Dut_C	hsp60		Kan, Hyg
Wt_Rec	pLL_Rec	N.A.	Recombination assay	Strep
Δloop-Dut_Rec	pLL_Rec; Δloop-dut_pGem, wt dut	dut; construct integrated to the genome		Strep, Gm
inactive-Dut_Rec	pLL_Rec; inactive-dut_pGem, dut::KO			Strep, Gm, Hyg

### Yeast two-hybrid screen

Yeast two-hybrid (Y2H) studies were performed by Hybrigenics Services (available in the public domain at www.hybrigenics-services.com, Paris, France). The coding sequences of the *M. tuberculosis* wt Dut protein (amino acids 1–154, accession number: Rv2697c) and the loop mutant Dut (Δloop-Dut) protein (132–137 amino acids deleted from Rv2697c) were cloned into the pB27 vector as C-terminal fusions to LexA using SfiI restriction sites to result in an N-LexA-dut-C fusion. The N-LexA-dut-C and N-LexA-Δloop-Dut-C constructs were quality-checked by sequencing and used as bait to screen 46.4 and 30.7 million clones of a *Mycobacterium tuberculosis*_genomic fragment DNA library (strain: H37Rv ref: MYT; Hybrigenics), respectively. A total of 333 and 175 colonies were selected on a medium containing 0.5 and 5 mM 3-amino-1,2,4-triazole (3-AT) for the wt and Δloop-Dut, respectively. The prey fragments of the positive clones were amplified by PCR and sequenced at their 5′ and 3′ junctions. The resulting sequences were identified in the GenBank database (NCBI) using a fully automated procedure. Each identified interaction was assigned a confidence score (predicted biological score [PBS]) in accordance with Hybrigenics criteria.

### Expression and purification of proteins in the study

The *M. tuberculosis* wt and nd AdnAB proteins were expressed and purified based on the protocol of [50]. The AdnA and AdnB polypeptides were co-expressed from separate pET19b and pET28a vectors in a BL21 DE3 pLysS strain of *E. coli* (Novagen, Merck) in LB medium. When the optical density (OD_600_) of the culture reached 0.4–0.6, protein expression was induced by 0.2 mM isopropyl-β-D-1-thiogalactopyranoside (IPTG) (Sigma), and incubation was continued overnight at 17°C. Cells were then harvested by centrifugation at 3440 *× g* for 20 min at 4°C. The cell pellet from 1 l culture was resuspended in 50 ml of lysis buffer containing 10% sucrose (Sigma), 50 mM TRIS pH 8 (Bio-Rad), 250 mM NaCl (Sigma), 0.1 mg/ml lysozyme (Sigma), 0.1% Triton-X100, and 2 mM β-mercaptoethanol (Sigma). Physical cell lysis was performed by sonication on ice using Bandelin Sonoplus HD 2070 sonicator (BANDELIN electronic GmbH & Co. KG; Berlin) at 60% power and 60% cycles for 4 × 1 min, followed by centrifugation (11 000 × *g*, 30 min, 4°C). The AdnAB heterodimer was purified from the supernatant using a Ni-NTA affinity column (Novagen), exploiting the N-terminal 10xHis tag on the AdnA. The column was equilibrated with the lysis buffer before protein binding took place. Subsequent washes were performed using buffer-A [10% glycerol (Thermo), 50 mM TRIS pH 8 (Bio-Rad), 250 mM NaCl (Sigma), 100 µM TCEP (Sigma), and 1 mM MgCl_2_ (Sigma)], and buffer-A supplemented with 40 mM imidazole (Sigma). Elution was performed with 200 mM imidazole in buffer-A. Affinity tags were removed by digestion with 0.1 mg TEV protease during overnight dialysis. To further purify and analyze the stoichiometry of the heterodimer, size-exclusion chromatography was carried out on a Superdex 200 10/300 GL (former GE) column equilibrated in buffer-A. Protein-containing fractions were analyzed by SDS–PAGE, and protein concentrations were determined by the BCA assay (Bio-Rad) or using a NanoDrop 2000c spectrophotometer (Thermo Scientific). The Superdex 200 10/300 GL column was calibrated with a suitable gel-filtration standard mixture (Bio-Rad #1 511 901).

The *M. tuberculosis* Dut was expressed and purified according to [60]. Briefly, wt and mutant Duts were expressed in the BL21 DE3 (Rosetta) strain of *E. coli* (Novagene, Merck) in LB medium from pet15b vectors for 4 h at 37°C following induction with 0.5 mM IPTG. The protein was purified using Ni-NTA affinity column (Novagen) exploiting the N-terminal His tag on the Dut.

### Isothermal titration calorimetry

Isothermal titration calorimetry (ITC) measurements were conducted using a MicroCal PEAQ-ITC instrument (Malvern). To prevent interference from buffer dilution, all proteins used in the experiments were dialyzed against the same buffer (10% glycerol [Thermo], 50 mM TRIS [Bio-Rad], 250 mM NaCl [Sigma], 100 µM TCEP [Sigma], 1 mM MgCl₂ [Sigma], pH 8). Measurements were performed at 37°C with a stirring speed of 750 RPM during titration. Injections were delivered at a mixing rate of 0.5 µl/s, with 150-s intervals between each injection. For experiments involving DNA, salmon sperm DNA (Invitrogen) with an average fragment size of 2 kb was used.

### Electrophoretic mobility shift assay

Oligonucleotides used for DNA-binding assays were labeled at their 5′ end with Cy3. The labeled strand was annealed to a longer, unlabeled complementary strand to generate a 5′-tailed double-stranded (dsDNA) substrate. Complex formation between dsDNA, nd AdnAB, and Dut was analyzed under the following conditions. Reaction mixtures (10 μl) contained 20 mM HEPES (pH 8.0), 50 mM NaCl, 1 mM MgCl₂, 10% (v/v) glycerol, 0.5 mM dithiothreitol (DTT), 0.1 μM 5′-Cy3-labeled dsDNA, 0.25 μM AdnAB, and increasing concentrations of Dut (0, 0.25, 0.5, 1, 2, 4, 8, and 16 μM). Reactions were incubated for 15 min at room temperature in the dark. Samples were resolved on a 10-cm native 6% polyacrylamide gel (19:1 acrylamide:bisacrylamide) prepared in 45 mM Tris-borate and 1.25 mM EDTA. Electrophoresis was performed at 120 V for 50 min in a cold room. Products were visualized by fluorescence imaging (Bio-Rad Chemidoc MP Imaging System).

### M-PFC assay

The trimethoprim (Tmp) resistance of M-PFC strains was assessed using 7H10 agar plates supplemented with 6 µg/ml Tmp. Strains were first inoculated into 7H9 liquid cultures containing the appropriate antibiotics. After overnight incubation, the cultures were diluted to an OD₆₀₀ of 0.3–0.4. A series of tenfold dilutions was then plated on both Tmp-containing and Tmp-free 7H10 agar plates. Plates were incubated at 37°C for 72 h to assess bacterial growth and resistance.

### Helicase assay

Helicase reaction mixtures (10 μl) contained 25 nM nd AdnAB, 0.1 μM 5′-Cy3-labeled 5′-tailed dsDNA substrate (described above), 10 μM unlabeled 24-mer DNA “trap” oligonucleotide (identical in sequence to the Cy3-labeled strand of the substrate), 2 mM ATP and 2.5 μM Dut or bovine serum albumin (BSA) in a buffer comprising 20 mM Tris–HCl (pH 8.0), 10 mM MgCl₂, 50 mM NaCl, and 0.5 mM DTT. Reaction mixtures were preincubated for 5 min at 37°C. Reactions were initiated by the addition of ATP + trap oligonucleotide. The trap oligonucleotide was included to prevent spontaneous reannealing of the unwound Cy3-labeled strand. Following initiation, samples were taken at different time points by the addition of 5 μl stop solution containing 245 mM EDTA, 0.5% (w/v) SDS, 30% (v/v) glycerol, and 375 μg/ml Orange G. A heat-denatured control was prepared by incubating the reaction mixture at 98°C for 3 min. Reaction products were resolved on a 10-cm native 14% polyacrylamide gel prepared in 45 mM Tris-borate, 1.25 mM EDTA, and 10% (v/v) glycerol. Electrophoresis was performed at 120 V for 120 min in a cold room. Products were visualized by fluorescence imaging and quantified by densitometry (BioRad Chemidoc MP Imaging System).

### dsDNase activity assay

The nuclease activity of AdnAB was measured following the modified method described by [50]. Briefly, proteins were pre-incubated with 20 ng/µl SmaI-digested pUC19 plasmid in 10 µl reaction volume in buffer-A (10% glycerol [Thermo], 50 mM TRIS [Bio-Rad], 250 mM NaCl [Sigma], 100 µM TCEP [Sigma], 1 mM MgCl₂ [Sigma], pH 8) at 37°C. The reaction contained 0.3 µM AdnAB and 11.3 nM linearized pUC19 plasmid DNA. Additional proteins or substrates were applied at the following concentrations: Dut 15 µM (concentration in monomers), dUPNPP 300 µM, BSA at a protein mass equivalent to that of 15 µM Dut (2.5 µg BSA/ reaction; 4 µM). The reactions were initiated by the addition of 1 mM ATP. Samples were taken at different time points and quenched by the addition of 5 μl stop solution containing 245 mM EDTA and 0.5% (w/v) SDS. Quenched samples were run on a horizontal native 1% agarose gel in TAE buffer (TRIS EDTA acetate). Gels were analyzed by densitometry.

### ATPase activity assay

The ssDNA-activated ATPase activity was measured using a coupled NADH oxidation assay. Reaction mixtures (250 μl) contained 20 mM Tris–HCl (pH 8.0), 10 mM MgCl₂, 50 mM NaCl, 1 mM phosphoenolpyruvate (PEP), 150 μM NADH, 2% pyruvate kinase–lactate dehydrogenase (PK–LDH) mixture (Sigma, P0294), 100 nM ssDNA (35 base length oligonucleotide), and 60 nM AdnAB. Reactions were monitored at 25°C using a Specord 200 UV/Vis spectrophotometer (Analytik Jena) and microvolume quartz cuvettes (Hellma 108.002F-QS). Absorbance at 340 nm was recorded continuously. After establishing a stable baseline, reactions were initiated by the addition of ATP to a final concentration of 1 mM. Data acquisition continued until the decrease in A340 reached a plateau. ATPase activity was calculated from the slope of the linear portion of the A340 time course.

### Recombination assay

The Rec-strains (Table 2) were grown in 3 ml of Lemco medium for 48 h at 37°C without antibiotic pressure to ensure that recombination events can take place. To simulate genotoxic stress, ciprofloxacin was supplemented into the growth medium at a final concentration of 0.3 µg/ml where specified. Colony-forming units (CFUs) were then determined on the following plates: antibiotic-free Lemco plate, Lemco plate containing Kan, and Lemco plate containing Kan + Strep. The plates were incubated at 37°C for 2 days. After incubation, CFUs were determined.

### Statistics

Non-linear curve fitting and statistical analyses were performed using the Origin 2018 software. Hypothesis testing was conducted using Student’s *t*-tests. In the dsDNase assay, reaction curves were fitted individually using an exponential decay function, then the obtained rate constants were compared to their respective pairs by performing two-sample *t*-tests. For the recombination assay, statistical significance between experimental groups was assessed via two-sample *t*-tests.

## Results

### Identification of AdnA as an interaction partner of *M. tuberculosis* dut using yeast two-hybrid screening

The Y2H screenings were performed by Hybrigenics Services, for which we provided the coding sequences of the full-length wt *M. tuberculosis* Dut (Dut, amino acids 1–154) and its Δloop mutant version as baits. The Δloop mutant lacks the 5-amino-acid-long mycobacterium-specific sequence (amino acids A133-S137, cf. Fig. 2 in [3]) that generates a surface loop on Dut. We previously found that the enzyme without this surface loop does not support viability even though it is active [3]. This suggests that the surface loop is essential for a non-catalytic function of Dut, possibly involving protein–protein interactions. Therefore, testing both the wt and Δloop versions allowed us to probe whether this specific structural element is required for interaction with partner proteins identified in the screen. The prey library was prepared from the *M. tuberculosis* genome. The Y2H screen identified various segments of the AdnA protein as the most likely binding partners for both the wt and the Δloop mutant Dut. All AdnA fragments that interacted with the bait contained the N-terminus of the protein (Fig. 2). The smallest fragment identified was an 85-amino-acid-long segment corresponding to the N-terminal portion of the AdnA DNA helicase ATP-binding subdomain (Fig. 2). No homologous segments of AdnB were identified in the screen. These results suggest that Dut interacts with the AdnAB heterodimer specifically through the N-terminus of AdnA.

### Design of AdnA fragments and production of the AdnAB heterodimer

Based on the Y2H fragment results shown in Fig. 2 and guided by structural considerations, we designed and expressed various *M. tuberculosis* AdnA protein fragments using an *E. coli* heterologous expression system to investigate the AdnA–Dut interaction. The constructs were carefully designed to preserve intact secondary structure elements, avoiding truncations within α-helices or β-sheets. Additionally, efforts were made to minimize unstructured residues at the C-terminus, thereby promoting proper folding and stability of the expressed protein fragments. The AdnA1-95 fragment was designed to represent the small, potentially stable fragment identified as a Y2H hit (Fig. 2). The AdnA1-229 fragment represents the 220 and 228-amino-acid-long hits in the Y2H screen (Fig. 2). Finally, the AdnA1-311 fragment was designed to represent the entire AdnA ATP binding domain. Although these AdnA fragments were successfully expressed, they were unstable and precipitated at various purification steps, preventing further biochemical characterization.

**Figure 3.**
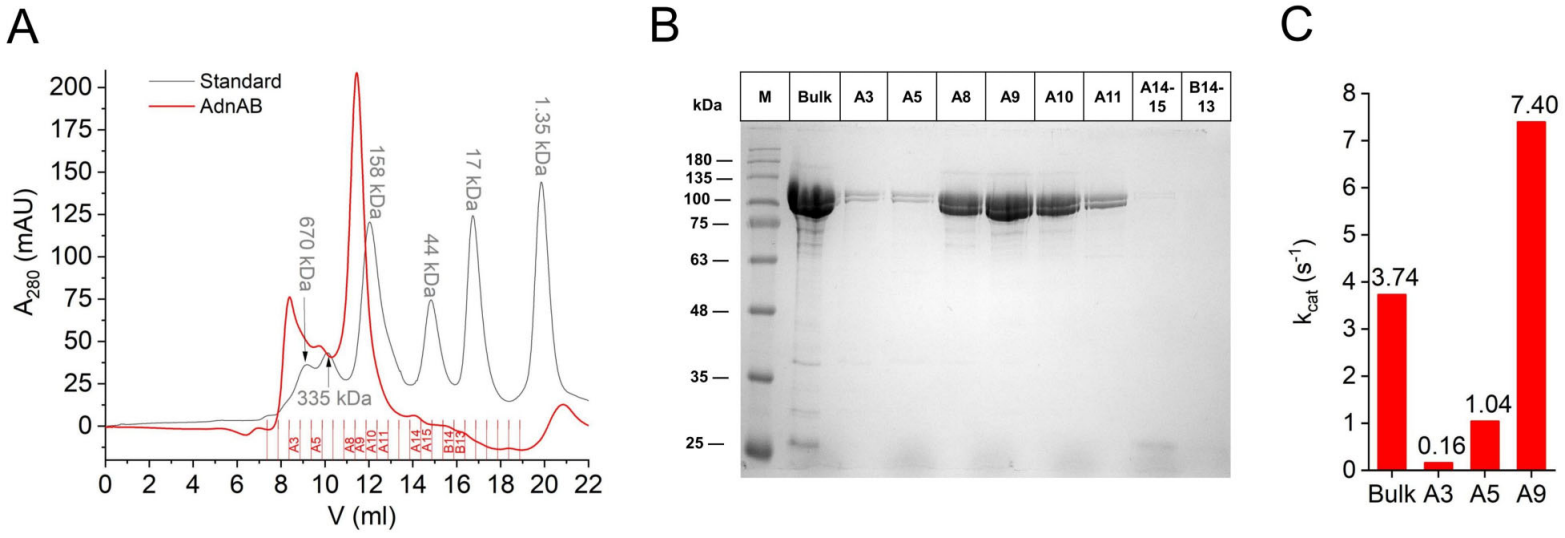
Assessment of the functional integrity of the *M. tuberculosis* AdnAB heterodimer. (**A**) Calibrated size-exclusion chromatography of tag-cleaved AdnAB. The elution profile (red) is shown relative to molecular weight standards (gray). Fractions analyzed by SDS–PAGE and ATPase assay are indicated. The major symmetrical peak between the 335 kDa and the 158 kDa standard peaks is consistent with the expected heterodimer at 230 kDa. (**B**) SDS–PAGE analysis of selected fractions from panel (A). Lane M, molecular weight marker (GRS Protein Marker MultiColour); Bulk, affinity-purified, tag-cleaved protein prior to gel filtration; A3, A5, A8–A11, A14–15, and B14–13, gel filtration fractions as indicated. AdnA and AdnB co-elute across the principal peak. (**C**) DNA-dependent ATPase activity of bulk protein and representative gel filtration fractions (A3, A5, and A9). Turnover numbers (*k*_cat_) were derived from NADH-coupled assays. The principal dimer fraction (A9) exhibits maximal activity, whereas aggregated material displays reduced activity.

We therefore opted to express the complete AdnAB heterodimer, as the orthologous complex from *M. smegmatis* had previously been successfully produced [50]. A comprehensive assessment of protein purity and quality was performed to ensure that the preparations were suitable for downstream biochemical analyses. Following affinity purification, removal of the His_10_ tag from AdnA was required to assess subunit stoichiometry because tagged AdnA (118 kDa) and untagged AdnB (117 kDa) are indistinguishable by SDS–PAGE (Supplementary Fig. S1). The affinity tag was removed by TEV protease digestion under optimized conditions to ensure complete and specific cleavage. The digestion mixture was then passed through the affinity column to remove uncleaved protein, the cleaved His₁₀ tag, and the His-tagged TEV protease, and the flow-through was subjected to size-exclusion chromatography. This analysis revealed a minor high-molecular-weight aggregate and a predominant symmetrical peak consistent with the expected ∼230 kDa AdnAB heterodimer (Fig. 3A). SDS–PAGE analysis of fractions across the elution profile confirmed that AdnA and AdnB co-elute in an approximately 1:1 stoichiometric ratio, indicating that the purified AdnAB complex is intact (Fig. 3B).

To further assess the functional integrity of the protein, DNA-dependent ATPase activity was measured for the bulk preparation prior to gel filtration and for representative fractions corresponding to the three distinguishable peaks in the chromatogram (A3, A5, and A9). The principal dimeric fraction (A9) displayed the highest activity (*k*_cat_ = 7.4 s⁻¹), whereas aggregated material showed markedly reduced activity. The bulk preparation exhibited intermediate activity, approximately half that of the purified heterodimer (Fig. 3C).

Given our extensive prior research on *M. tuberculosis* Dut, this latter protein was readily available for further experiments (Supplementary Fig. S1).

### Validation of the AdnAB-dut protein–protein interaction

#### *In vitro* validation using isothermal titration calorimetry

**Figure 4.**
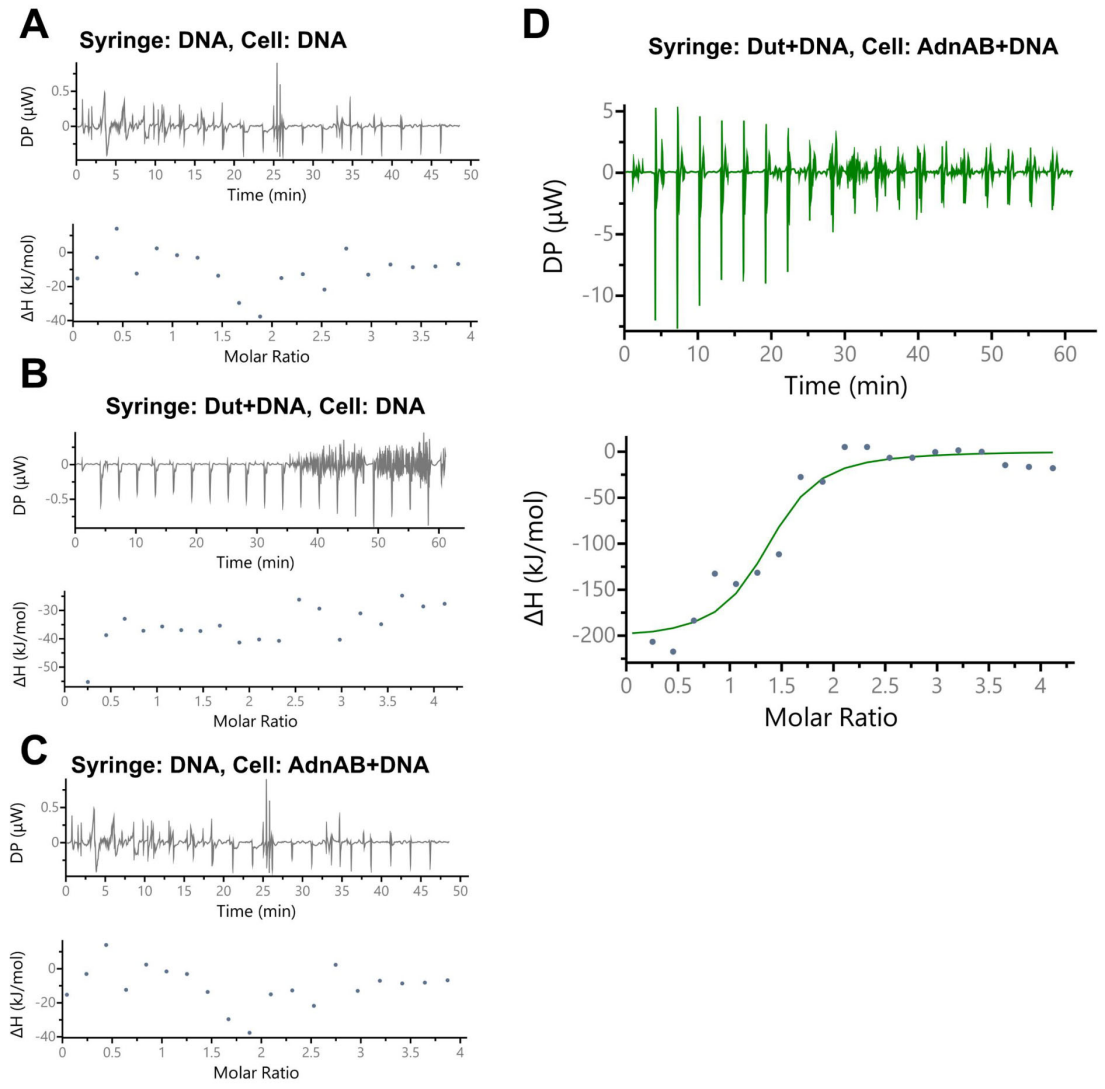
Quantification of the AdnAB–Dut interaction by ITC. (**A**) Control titration showing heat changes upon mixing 2.5 mg/ml sonicated salmon sperm DNA (average fragment size ∼2 kb). (**B**) Control titration of 78.8 µM Dut (monomer concentration) in buffer A supplemented with 2.5 mg/ml DNA into buffer A with the same DNA concentration, confirming that Dut alone does not generate binding heats with DNA. (**C**) Control titration of 2.5 mg/ml DNA in buffer A into 3.94 µM AdnAB supplemented with DNA, verifying that AdnAB was saturated with DNA and that no additional heat signal arises from AdnAB–DNA binding. (**D**) Experimental titration of 78.8 µM Dut (monomer concentration) into 3.94 µM AdnAB, both in buffer A supplemented with 2.5 mg/ml DNA. The resulting isotherm was fitted with a one-to-one binding model. Fitted parameters: *K*_d_ = 0.190 ± 0.126 µM; Δ*G* = −39.9 kJ/mol; Δ*H* = −206 ± 21.5 kJ/mol; −*T*Δ*S* = 166 kJ/mol; *N* (binding sites) = 1.31 ± 0.078 mol/mol.

We used ITC measurements to characterize the interaction between the purified AdnAB heterodimer and Dut. Measurements were performed at 37°C with AdnAB in the cell and Dut in the syringe. In the absence of DNA, no detectable interaction was observed (Supplementary Fig. S2). In contrast, when sonicated salmon sperm DNA was included in both the syringe (Dut) and the cell (AdnAB), a clear binding signal was obtained (Fig. 4). Control experiments excluded possible artifacts: DNA mixing (Fig. 4A) and Dut dilution (Fig. 4B) produced negligible heat changes, and AdnAB was verified to be saturated with DNA so that the observed heats could not arise from AdnAB–DNA interactions (Fig. 4C). The resulting Dut–AdnA binding isotherm (Fig. 4D) was well fit by a one-to-one binding model, considering the AdnAB heterodimer and the Dut monomer as the interacting units. The resulting dissociation constant (*K*_d_) is 0.19 ± 0.13 μM. The thermodynamic parameters (Δ*H*= −206 kJ/mol; −T*Δ*S* = 166 kJ/mol) indicate that the interaction is predominantly enthalpy-driven, although the entropic component also contributes significantly to the overall binding free energy.

#### *In vitro* validation using electromobility shift assay

A nuclease-dead variant of AdnAB (nd AdnAB) was generated to enable DNA-binding assays without confounding DNA degradation. We initially constructed the AdnA D935A–AdnB D1014A mutant heterodimer, targeting conserved catalytic residues within the nuclease domains based on [51]. However, the AdnA D935A variant proved highly unstable during expression and purification, yielding insufficient soluble protein for biochemical analysis. To overcome this limitation, we successfully generated an alternative substitution, D935N, which represents a structurally more conservative change while maintaining loss of nuclease activity (Supplementary Fig. S1). The AdnA D935N–AdnB D1014A mutant was therefore used in all subsequent electromobility shift assay (EMSA) and helicase experiments.

**Figure 5.**
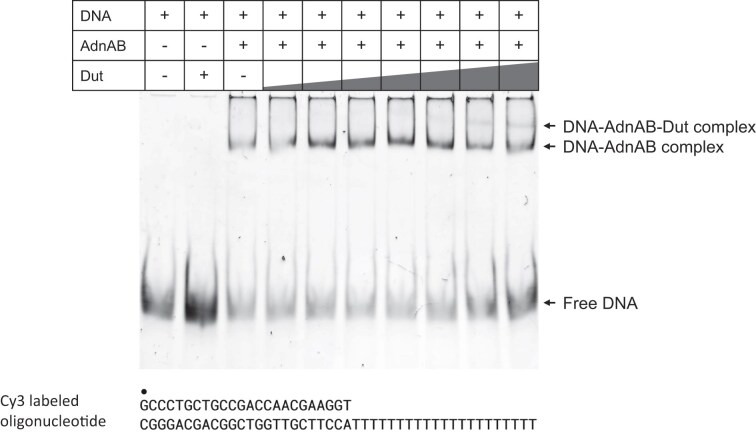
*In vitro* validation of the AdnAB-Dut interaction using EMSA. Reaction mixtures (10 μl) containing 0.1 μM fluorescently labeled DNA substrate, 0.25 μM nd AdnAB, and increasing concentrations of Dut (0, 0.25, 0.5, 1, 2, 4, 8, and 16 μM) were incubated for 15 min at room temperature in the dark. The samples were analyzed on a 10–cm native 6% polyacrylamide (19:1) gel in a cold room. The DNA substrate is shown at the bottom, with Cy3 label denoted by •. The EMSA was repeated several times.

Ternary complex formation between nd AdnAB, dsDNA, and Dut was examined using EMSA with a fluorescently labeled dsDNA substrate (Fig. 5). The concentrations of nd AdnAB and dsDNA were kept constant while increasing amounts of Dut were added. Dut alone did not bind DNA, whereas nd AdnAB formed a stable protein–DNA complex that produced a pronounced mobility shift. At higher Dut concentrations, a further mobility shift appeared, consistent with formation of a ternary AdnAB–Dut–DNA complex. Control reactions lacking either protein confirmed that the observed shifts were specific and that Dut alone does not interact detectably with DNA (Fig. 5).

Together, these results provide direct biochemical evidence that Dut associates with AdnAB and modulates its DNA-binding activity *in vitro*.

#### *In vivo* validation using the mycobacterial protein fragment complementation (M-PFC) assay

**Figure 6.**
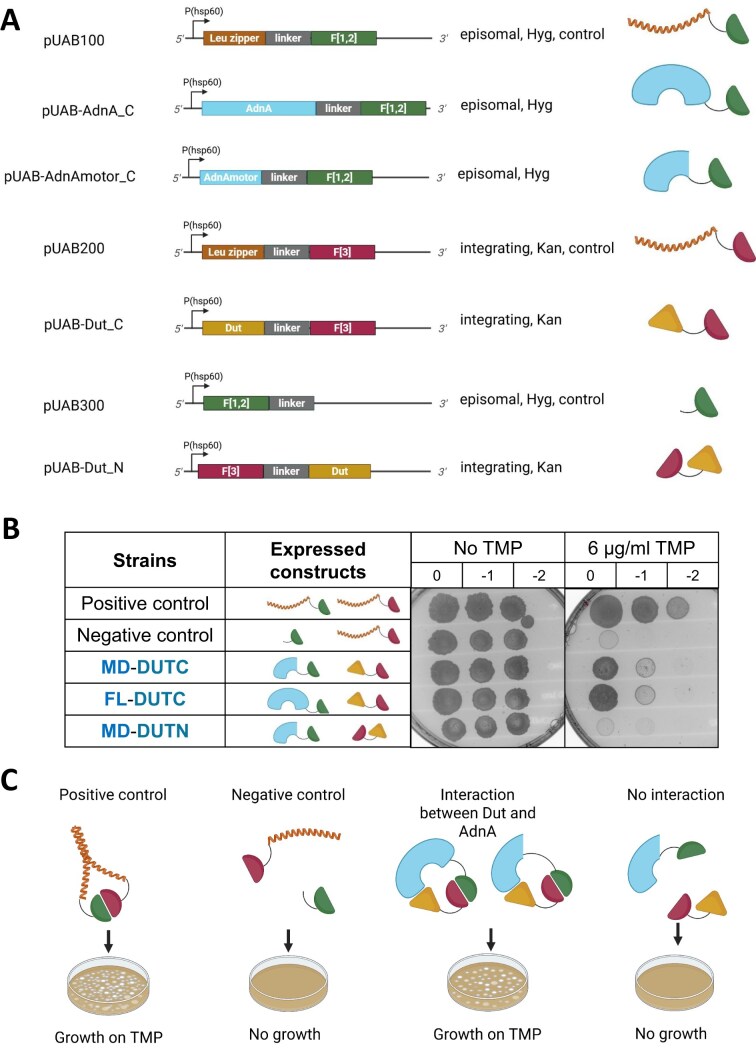
The AdnA–Dut interaction supports bacterial growth in an M-PFC assay. (**A**) DNA constructs used in the complementation assay. (**B**) Viability of the different strains in non-selecting and selecting media containing Tmp. Cells were applied in different dilutions. 0 denotes the undiluted culture; -1 and -2 denote 10× and 100× dilutions, respectively. The experiment was repeated three times. (**C**) Interpretation of the results. The AdnA–Dut interaction enables the reconstruction of the murine mDHFR which complements the inhibitory effect of Tmp. MD, AdnA motor domain; FL, full-length AdnA; DUTC, Dut fused to DHFR at its C-terminus; DUTN, Dut fused to DHFR at its N-terminus. Created in BioRender. Toth, J. (2026) https://BioRender.com/nescxis.

Using a previously developed assay aimed at contributing to the systematic assembly of mycobacterial protein interaction maps [56], we investigated the *in vivo* association between mycobacterial AdnA and Dut in *M. smegmatis*. For this assay, various AdnA constructs and the *dut* gene were independently cloned into vectors, which encode complementary domains of murine dihydrofolate reductase (mDHFR) represented by green and red half-circles in Fig. 6. The recombinant vectors shown in Fig. 6A and Table 1 were then co-transformed into *M. smegmatis* in combinations shown in Fig. 6B. In this assay, a successful *in vivo* protein–protein interaction restores functional mDHFR in co-transformants, enabling their survival on media containing trimethoprim (Tmp) at otherwise inhibitory concentrations (Fig. 6C). In our experiments, the controls behaved as expected: the positive control, containing a Leu zipper (orange helix in Fig. 6) supported robust cell growth, whereas the negative control, expressing only one interaction partner of the coiled-coil pair in the Leu zipper, did not grow (Fig. 6B). We observed cell growth in AdnA–Dut co-transformants depending on the configuration of Dut (yellow triangle in Fig. 6) with the mDHFR fragment. When the mDHFR fragment was fused to the C-terminus of Dut (yellow triangle linked to red half-circle to its right in Fig. 6), strains transformed with either full-length AdnA (blue horseshoe) or the AdnA motor domain (blue half-horseshoe in Fig. 6) exhibited similar cell growth, supporting the interaction between *M. tuberculosis* AdnA and *M. tuberculosis* Dut in *M. smegmatis*. However, no cell growth was observed when the mDHFR fragment was fused to the N-terminus of Dut (yellow triangle linked to red half-circle to its left, Fig. 6B and C), indicating a role of the Dut N-terminus in the AdnA–Dut interaction. Negative control experiments, where target proteins fused to one fragment of mDHFR were combined with the complementary fragment without the interacting partner, are shown in Supplementary Fig. S3.

### The nuclease activity of AdnAB increases in the presence of structurally intact and active dut

**Figure 7.**
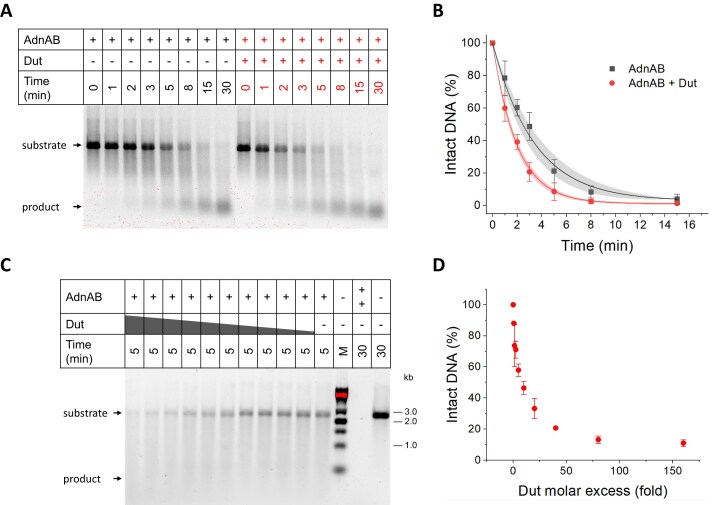
Dut activates the AdnAB nuclease activity in a concentration-dependent manner. (**A**) Nuclease activity of AdnAB with (red) or without (black) the addition of Dut. 0.3 µM AdnAB was mixed with 15 µM Dut and 11.3 nM (20 ng/μl) linearized pUC19 plasmid DNA, and the reaction was started with the addition of 1 mM ATP. Samples were collected at the indicated time points and analyzed by gel electrophoresis in a 1% agarose gel. (**B**) Time courses of the data extracted from the gels in panel (A) by densitometry, normalized to *t* = 0. The measurement was performed in triplicate; the error bars indicate SD. The *y* = *A*_1_*exp(−x*k) + *y*_0_ function was fitted to the data (black and red lines), which yielded the apparent rate constants *k* = 0.30 ± 0.020 min^−1^ for AdnAB, and *k* = 0.51 ± 0.015 min^−1^ for AdnAB + Dut. The 95% confidence intervals are shown as shaded regions around the fitted curves. Differences in the derived rate constants are statistically significant at the *P* = 0.05 level. (**C**) Dut concentration dependence of the nuclease activity of AdnAB. The reaction contained 0.3 µM AdnAB and 11.3 nM linearized pUC19 plasmid DNA with Dut at various concentrations (indicated in panel D). The positive control (++) contained DNase I (NEB) in addition to AdnAB, while the negative control did not contain any enzymes. “M” designates the GRS Ladder 1 kb DNA marker. (**D**) Dut titration curve of the AdnAB activity change obtained by densitometry of the gels in panel (C). Data are normalized to c(Dut) = 0. Measurements were performed in triplicate; error bars represent standard deviations.

After confirming the AdnAB-Dut interaction both *in vitro* using purified proteins, and in the mycobacterial cell, we investigated its potential functional consequences on the AdnAB nuclease activity. In the *in vitro* nuclease assay, we used linearized plasmid DNA as a well-defined substrate for AdnAB, containing two dsDNA ends per molecule. We premixed the DNA with the proteins, allowing any complex formation to take place, and started the reaction with the addition of 1 mM ATP, the substrate for AdnAB but not for Dut. AdnAB cleaved DNA 1.7-fold faster in the presence of Dut, a difference that was statistically significant (*P* = 4.65 × 10^-4^), as shown by the time courses in Fig. 7A and B. This nuclease-enhancing effect was dependent on Dut concentration (Fig. 7C and D). The relationship did not conform to a simple hyperbolic or quadratic function, suggesting that the underlying mechanism is more complex.

**Figure 8.**
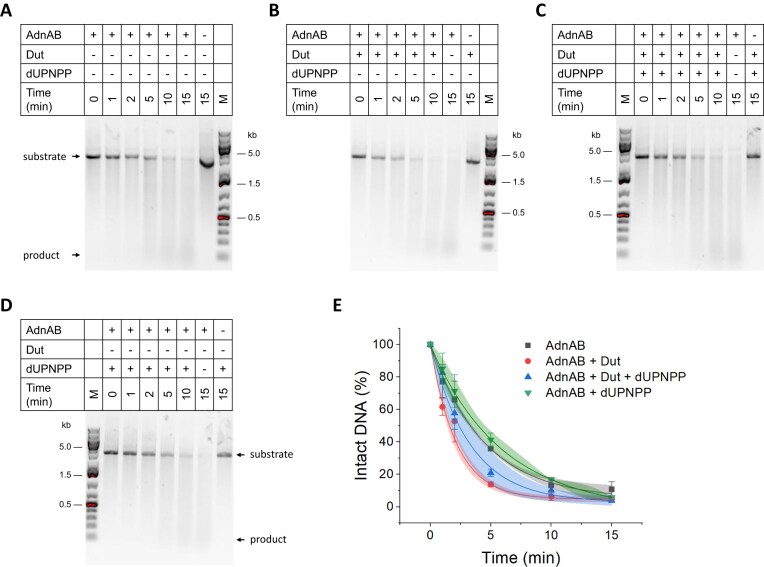
The substrate analog of Dut partially disrupts AdnAB nuclease activation. Nuclease activity of 0.3 µM AdnAB on 11.3 nM linearized pUC19 plasmid DNA in the absence (**A**) and the presence of 15 µM Dut (**B**), 300 µM dUPNPP (**D**), or both (**C**). The reaction was started with the addition of 1 mM ATP. Samples were taken at the time points indicated and then analyzed by gel electrophoresis in 1% agarose gel. “M” designates the GeneRuler 1 kb DNA Ladder (Thermo Scientific). (**E**) Time courses of the data extracted from the gels in panels (A–D) by densitometry, normalized to *t* = 0. The measurement was done in triplicate; the error bar indicates SD. The *y* = *A*_1_*exp(−x*k) + *y*_0_ function was fitted to the data (continuous lines), yielding the apparent rate constants *k* = 0.23 ± 0.011 min^−1^ for AdnAB, *k* = 0.47 ± 0.024 min^−1^ for AdnAB + Dut, *k* = 0.30 ± 0.036 min^−1^ for AdnAB + Dut + dUPNPP, and *k* = 0.17 ± 0.0006 min^−1^ for AdnAB + dUPNPP. The 95% confidence intervals are shown as shaded areas around the fitted curves. Statistically significant differences in the derived rate constants (*P* < 0.05) were observed only for the AdnAB + wt Dut condition without nucleotide, compared to AdnAB alone.

We investigated whether the substrate binding of Dut influences its functional interaction with AdnAB. We used 300 µM of the quasi-non-hydrolyzable substrate analog dUPNPP in order to create a well-defined nucleotide-bound conformational state of the Dut enzyme within the reaction mixture. The addition of dUPNPP in the same reaction mixture as in Fig. 7 resulted in a decrease in the enhancement of the AdnAB nuclease activity (red versus blue time course in Fig. 8). Statistical analysis indicates that the derived rate constant in the presence of dUPNPP is not significantly different from AdnAB alone, while showing a marginal difference compared to AdnAB + Dut (*P* = 0.053; Supplementary Table S2).

**Figure 9.**
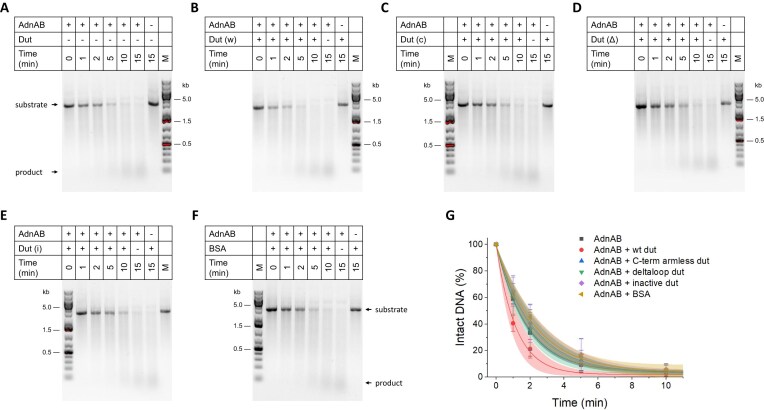
Dut mutations alter the effect of Dut on AdnAB nuclease activity. Nuclease activity of 0.3 µM AdnAB on 11.3 nM linearized pUC19 plasmid DNA in the absence (**A**) and in the presence of 15 µM wt Dut, Dut(w) (**B**); 15 µM C-terminally truncated Dut, Dut(c) (**C**); 15 µM Δloop Dut, Dut(Δ) (**D**); 15 µM catalytically inactive but structurally intact Dut, Dut(i) (**E**); or BSA (at a protein mass equivalent to that of Dut, 4 µM BSA) as a control (**F**). The reaction was started with the addition of 1 mM ATP. Samples were collected at the indicated time points and analyzed by gel electrophoresis in a 1% agarose gel. “M” designates the GeneRuler 1 kb DNA Ladder (Thermo Scientific). (**G**) Time courses of the data extracted from the gels in panels (A–D) by densitometry, normalized to *t* = 0. The measurement was performed in triplicate; the error bars indicate SD. The *y* = *A*_1_*exp(−x*k) + *y*_0_ function was fitted to the data (continuous lines), yielding the apparent rate constants *k* = 0.56 ± 0.04 min^−1^ for AdnAB, *k* = 0.87 ± 0.09 min^−1^ for AdnAB + wt Dut, *k* = 0.47 ± 0.03 min^−1^ for AdnAB + C-term armless Dut, *k* = 0.49 ± 0.04 min^−1^ for deltaloop Dut, *k* = 0.44 ± 0.03 min^−1^ for inactive Dut, and *k* = 0.44 ± 0.04 min^−1^ for BSA control. The 95% confidence intervals are shown as shaded areas around the fitted curves. Differences in the derived rate constants are statistically significant at *P* < 0.05 for all reactions when compared to the AdnAB + wt Dut condition (cf. Supplementary Table S2).

We also assayed various Dut mutants previously used in our *in vivo* enzymology experiments [61, 62, 3]. These mutants are either inactive or missing an important structural feature. None of these mutants could accelerate the dsDNA nuclease activity of AdnAB (Fig. 9). Nor did BSA used for non-specific protein binding control (Fig. 9). The derived rate constants were statistically indistinguishable from those of the AdnAB reaction alone, yet significantly different from the enhancement observed with wt Dut.

### Dut does not enhance the intrinsic helicase activity of AdnAB

**Figure 10.**
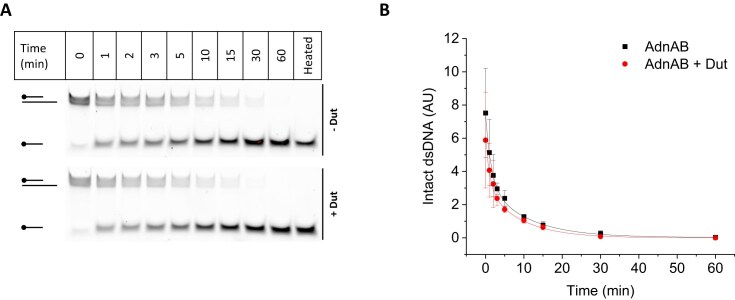
Dut increases the formation of the population of active helicase complexes. (**A**) Representative helicase assay. Reaction mixtures containing 25 nM nd AdnAB, 100 nM Cy3-labeled 5′-tailed duplex DNA substrate, and 2.5 μM Dut or BSA were preincubated for 5 min at 37°C. Reactions were initiated by the addition of 2 mM ATP mixed with 10 μM unlabeled trap DNA and incubated for 60 min at 37°C before quenching with 5 μl stop solution. A heat-denatured control was prepared by incubating the reaction mixture at 98°C for 3 min. The DNA substrates are shown below the gel; the Cy3 label is indicated by •. (**B**) Time courses derived from densitometric analysis of the assay gels. Signal intensities were not normalized to avoid unnecessarily distorting the time courses. Measurements were performed in duplicate, and error bars indicate SD. The data were fitted to a double-exponential decay function (continuous lines), yielding the following parameters: *k*₁ = 0.67 ± 0.13 min⁻¹ and *k*₂ = 0.094 ± 0.011 min⁻¹ for AdnAB; and *k*₁ = 0.91 ± 0.38 min⁻¹ and *k*₂ = 0.11 ± 0.009 min⁻¹ for AdnAB + DUT. The differences between corresponding rate constants were not statistically significant.

Using the nd AdnAB, we could study the effect of the Dut interaction on the separated helicase function of the enzyme. In the helicase assay, a Cy3–labeled 5′–tailed duplex oligonucleotide substrate was used together with an unlabeled 24–mer “trap” oligonucleotide, identical to the labeled strand of the substrate, to prevent spontaneous reannealing of the displaced labeled strand (Fig. 10A).

Reactions were initiated by the simultaneous addition of ATP and trap DNA to preincubated AdnAB–substrate or AdnAB–substrate–Dut complexes, and the time course of DNA unwinding was monitored by gel electrophoresis (Fig. 10A). Densitometric quantitation of the gel bands followed by kinetic analysis revealed no detectable effect of Dut on the unwinding kinetics (Fig. 10B).

### Perturbation of dut function reduces recombination frequency in *M. smegmatis*

**Figure 11.**
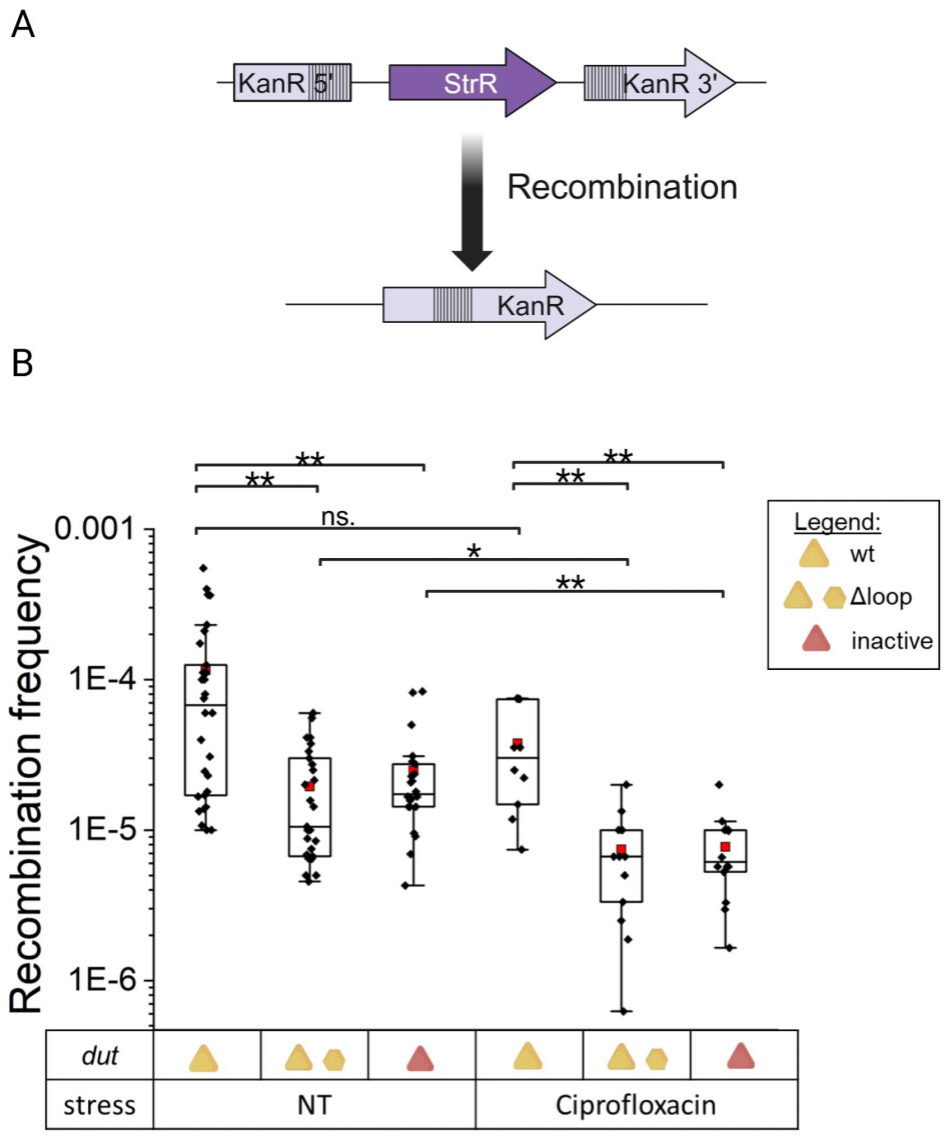
Results of the recombination frequency assay. (**A**) Schematic representation of the recombination event occurring within the experimental system. The shaded regions indicate sequence homology. (**B**) Recombination frequency detected in the various Dut perturbed strains. The box represents the interquartile range (IQR), spanning from the 25th percentile (Q1) to the 75th percentile (Q3). The horizontal line inside the box indicates the median (50th percentile). The whiskers extend from the box to the smallest and largest values within 1.5 × IQR from Q1 and Q3, respectively. The mean is represented by a red square. Means and SD are as follows: (1.15 ± 1.4) × 10^−4^ for the wt, (1.95 ± 1.6) × 10^−5^ for the wt + Δloop, and (2.48 ± 2.1) × 10^−5^ for the inactive strain in non-treated (NT) conditions; and (3.76 ± 2.7) × 10^−5^ for the wt, (7.44 ± 5.3) × 10^−6^ for the wt + Δloop, and (7.73 ± 4.7) × 10^−6^ for the inactive strain in ciprofloxacin treated conditions. “ns” denotes non-significant change at a *P* = 0.05 level; “*” denotes significant change at a *P* = 0.05 level; “**” denotes significant change at a *P* = 0.01 level.

We investigated how perturbing Dut function affects homologous recombination frequency in our *M. smegmatis* system. In addition to the wild-type strain (yellow triangles in Fig. 11), we analyzed two Dut mutant strains under stress-free conditions and following exposure to genotoxic stress induced by ciprofloxacin. The *dut*(i) strain is a complemented *dut* knock-out in which the complementing copy is a structurally intact but inactive Dut (*dut*^−^/*dut*^i^), shown as a red triangle in Fig. 11 [3]. The Δloop *dut* mutant is a merodiploid strain carrying both the wt and a Δloop mutant Dut lacking a specific five-amino-acid surface loop characteristic of all mycobacterial Duts (*dut*^+^/*dut*^loop^) [3]. This mutant protein (depicted as a chopped yellow triangle in Fig. 11) retains an otherwise intact structure [60] and has only slightly reduced enzymatic activity. However, it cannot complement the lethal phenotype of a Dut knock-out, so it can only be maintained in a merodiploid state [3].

To investigate the potential role of Dut in DSB repair, we assessed the above two mutant strains and the wt strain under both untreated conditions and following induction of DSBs through genotoxic stress mediated by gyrase inhibition with ciprofloxacin.

To quantify recombination frequency, we used a previously described assay [63] modified to suit our antibiotic selection markers. As illustrated in Fig. 11A, the assay is based on an antibiotic resistance switch: homologous recombination between two split kanamycin resistance gene segments (KanR) results in the loss of streptomycin resistance (StrR) and the gain of kanamycin resistance.

Both Dut mutant strains exhibited a 3.5–5-fold significant decrease in recombination frequency compared to the wild-type strain. When the experiment was repeated under genotoxic stress induced by ciprofloxacin [64], homologous recombination efficiency in the mutant strains was further reduced (Fig. 11B).

## Discussion

This study identifies a previously unrecognized interaction between *M. tuberculosis* Dut and the AdnAB DNA repair complex, revealing an unexpected crosstalk between nucleotide metabolism and DNA double-strand break repair. Using a multifaceted approach that integrates yeast two-hybrid screening, biophysical assays, *in vivo* validation, and functional analyses, we demonstrate that Dut physically associates with the AdnAB complex and enhances its nuclease activity *in vitro*. In parallel, Dut functional mutations impact recombination efficiency *in vivo*. Together, these observations point to a potential connection between nucleotide metabolism and the machinery responsible for DNA double-strand break processing in mycobacteria.

### Interaction between Dut and AdnAB

Our experiments show that Dut can bind to the AdnAB complex, the primary ATP-dependent end resection enzyme in mycobacteria [50], and modulate its activity *in vitro*. ITC measurements indicated that the AdnAB–Dut interaction is DNA-dependent, a result that was further supported by EMSA. At elevated Dut concentrations, additional DNA–protein complexes were observed that were not present in the absence of Dut. Although the exact composition of these species cannot be determined from these experiments, their appearance is consistent with the formation of higher-order complexes containing AdnAB, DNA, and Dut.

### Mechanistic insight from helicase and nuclease assays

In nuclease assays, the presence of Dut increased the apparent rate of DNA degradation by AdnAB, although the magnitude of this stimulation remained modest (Fig. 7). The effect was reproducible and statistically significant across independent measurements. Mutations that compromise the structural integrity of Dut, including catalytic inactivation or deletion of the mycobacteria-specific loop, reduced this stimulatory effect (Fig. 9).

The nucleotide-bound state of Dut also influenced this behavior. When Dut was stabilized in the substrate-bound conformation using the non-hydrolyzable analog dUPNPP, the enhancement of AdnAB nuclease activity was reduced (Fig. 8). These observations suggest that the conformational state of Dut influences its interaction with the AdnAB complex.

Helicase assays performed with a nuclease-dead AdnAB variant revealed no detectable difference between reactions carried out in the presence or absence of Dut, indicating that Dut does not affect the observed unwinding kinetics (Fig. 10). Therefore, the increased rate of DNA degradation observed in nuclease assays is likely to originate from a step other than strand separation. Possible explanations include an effect of Dut on AdnAB–DNA interaction dynamics or on the nuclease activity of the complex itself. Further experiments will be required to discriminate between these mechanisms.

### Structural determinants of the interaction on the dut side

Our previous work showed that intact Dut is essential for mycobacterial viability, and that deleting the mycobacterium-specific loop impairs double-crossover (DCO) recombination [3]. Structural analysis of the Δloop mutant revealed that, apart from altered anchorage of the C-terminus—a region crucial for trimer stability and catalytic dynamics—its overall fold is preserved [60], suggesting a role in protein–protein interactions rather than catalysis.

To elucidate why both mutants exert comparable effects in the *in vitro* and the recombination assays, we can draw on insights from the only well-characterized Dut–protein interaction, with the Stl repressor. Mutations affecting the C-terminal arm (conserved motif V) in this system lead to profound functional loss [30, 65, 66]. Both mutants in our study disrupt the conformational equilibrium of this region [60, 66], and the same Δloop mutant has been shown to be hypofunctional in Stl inhibition experiments [60]. Together, these observations suggest that mutations compromising a fully functional C-terminal arm impair AdnAB enhancement, even if binding can still be detected in Y2H assays. This may also explain why the nucleotide-bound state of Dut, locked by dUPNPP, fails to enhance AdnAB nuclease activity: the C-terminal arm is trapped in a predominant conformation, preventing functional activation.

### Structural determinants on the AdnAB side

The interaction with Dut appears to involve the AdnA subunit of the complex. No interaction with AdnB was detected, consistent with the asymmetric architecture of the AdnAB heterodimer. Within AdnA, the smallest interacting fragment identified corresponds to the N-terminal 85 amino acids, encompassing the helicase ATP-binding subdomain. This region of AdnA has remained functionally enigmatic. Although it contains recognizable Walker A and Walker B motifs, ATP hydrolysis activity has not been detected for this domain [51]. The observation that this region can interact with Dut suggests that it may participate in protein–protein interactions that influence AdnAB activity or DNA engagement.

It is worth noting that the orthologous *M. smegmatis* AdnAB has been reported to unwind and degrade DNA at much faster rates, suggesting it is considerably more active than we observed. Importantly, the structural and functional integrity of our *M. tuberculosis* AdnAB heterodimer has been carefully validated (Fig. 2). Those previous measurements were obtained using the *M. smegmatis complex*, whereas the present study focuses on the orthologous enzyme from *M. tuberculosis*. While the two proteins are closely related, differences in the stability of the heterodimer and of the AdnA D935A point mutant during protein purification suggest real structural variations.

### Biological context

The relative abundance of dUTPase and AdnAB *in vivo* indicates that the high dUTPase:AdnA stoichiometry used in our assays reflects physiologically relevant conditions. According to the PaxDb Protein Abundance Database, AdnA is expressed at exceptionally low levels in *M. tuberculosis* H37Rv, placing it among the lowest 5% of quantified proteins (Supplementary Fig. S4). In contrast, Dut ranks in the top quartile of the proteome, indicating a large difference in protein abundance relative to AdnA under stress-free conditions (Supplementary Fig. S4). Upon genotoxic stress, Molnár *et al*. [64] reported that exposure to double-strand break-inducing agents such as mitomycin C or ciprofloxacin elicits an 8–14-fold increase in AdnA mRNA levels, consistent with activation of homologous recombination repair pathways.

In the *in vivo* protein interaction M-PFC assay, both the motor and full-length AdnA constructs supported cell growth when co-expressed with Dut, whereas negative controls showed no growth (Fig. 6). The ability to detect even weak growth is remarkable, given that detection depends on sustained protein–protein interactions to maintain DHFR expression, which provides essential metabolic functions in the cell. The DHFR fragment N-terminally fused to Dut did not support growth, suggesting that the molecular surface comprising the N-terminus participates in the interaction with AdnA.

The interaction between Dut and AdnAB is DNA-dependent, and since AdnAB preferentially binds to DNA with free ends, this suggests that their interaction may be facilitated at sites where DNA ends are exposed, such as at double-strand breaks or during DNA repair.

### *In vivo* effects on recombination

Cells expressing inactive Dut exhibited a reduced recombination frequency, suggesting a defect in DNA processing. This phenotype aligns with our previous findings in *M. smegmatis*, where inactivation of Dut, unlike the bifunctional dCTP deaminase–Dut, led to increased mutation rates, despite both causing dNTP pool imbalances [34]. These observations indicate that genome instability in Dut-deficient cells is not solely due to nucleotide imbalance or uracil misincorporation but may involve a disruption of DNA repair processes, potentially through the loss of interaction with AdnAB.

Importantly, increased uracil incorporation into DNA is typically associated with *increased* recombination frequency. For example, *Arabidopsis* mutants with reduced Dut activity accumulate uracil in DNA and exhibit elevated homologous recombination [23]. Similarly, uracil-containing λ DNA promotes recombination even in non-replicating contexts [67]. In contrast, in our system, uracil accumulation induced by Dut inhibition due to a mutation correlated with a *decrease* in recombination frequency. This further supports the idea that uracil incorporation alone does not account for the reduced recombination observed in our Dut-perturbed strains.

The fact that the Δloop mutant retains enzymatic activity yet still exhibits reduced recombination reinforces the conclusion that Dut contributes to homologous recombination through a structural or protein–protein interaction-based role, independent of its catalytic function in uracil prevention.

### Possible functional implications

Although AdnAB is a central component of HR in mycobacteria, it is not essential under normal growth conditions. In AdnAB-deficient strains, HR activity is reduced by approximately 50%, yet viability is maintained [47], indicating the notorious high degree of functional redundancy in the mycobacterial DNA repair network. This redundancy complicates mechanistic dissection, as multiple pathways can compensate for one another under different conditions [68]. Our data suggest that the interaction between Dut and AdnAB may contribute to the modulation of HR, particularly under DNA-damaging stress. We have previously shown that ciprofloxacin and mitomycin C exposure, which induce DSBs, trigger robust upregulation of AdnAB expression [64], reinforcing its prominent role in DSBR under stress. Given that HR is the only high-fidelity repair pathway available for such lesions, its proper regulation is essential for genome maintenance.

Intriguingly, while Dut activity per se is not essential, since it can be functionally replaced by a bifunctional dCTP deaminase–Dut enzyme, the presence of a structurally intact Dut protein remains indispensable [34]. This paradox suggests one or more non-enzymatic roles for Dut.

Within this context, the interaction between Dut and AdnAB described here may represent one of several factors that influence the efficiency of DNA double-strand break processing. Our biochemical experiments indicate that Dut can facilitate the AdnAB nuclease activity *in vitro*, while genetic experiments suggest that perturbation of Dut affects recombination frequency *in vivo*. Although these observations are consistent with a functional connection between the two proteins, additional studies will be required to establish the physiological relevance and mechanistic basis of this interaction in greater detail.

Finally, these findings add to growing evidence that metabolic enzymes can participate in cellular processes beyond their primary catalytic roles. Whether the interaction between Dut and AdnAB contributes to coordination between nucleotide metabolism and DNA repair under specific physiological conditions remains an open question.

## Supplementary Material

gkag660_Supplemental_File

## Data Availability

The data underlying this article are available in Figshare at https://doi.org/10.6084/m9.figshare.28930187.
