# Supplementary material for: dUTPase modulates mycobacterial homologous recombination and interacts with the AdnAB helicase–nuclease
Source: Nucleic Acids Res. 2026 Jun 27;54(12):gkag660. doi: 10.1093/nar/gkag660 (PMC13309787; doi:10.1093/nar/gkag660)
Supplement: gkag660_Supplemental_File [file gkag660_supplemental_file.pdf]

SUPPLEMENTARY MATERIAL FOR

dUTPase Modulates Mycobacterial Homologous Recombination and Interacts with the AdnAB helicase-nuclease

Rita Hirmondó[1#], Dániel Molnár[1#], Gergely Döbrőssy[1, 2], Szonja T. Kovács[1], Beáta G. Vértessy[1,3]* and Judit Tóth[1,]*

[1] Institute of Molecular Life Sciences, HUN-REN Research Centre for Natural Sciences, Budapest, 1117, Hungary
[2]Molecular Medicine Division, Doctoral College, Semmelweis University, Budapest, 1085, Hungary
[3] Department of Applied Biotechnology and Food Science, Budapest University of Technology and Economics, Budapest, 1111, Hungary

[#] authors contributed equally

* To whom correspondence should be addressed. Tel: +36 1 382 6793; Email: toth.judit@ttk.hu

Correspondence may also be addressed to Tel: +36 1 382 6707; Email: vertessy.beata@ttk.hu

Supplementary Table 1: Primers used in the study

Name of primer	Sequence	Usage
AdnA_*D935A*	5’- GTTATTGTGG*C*TATTAAGACCGGTAAAACCCCGGTGAGCAAAGATGATGCC -3’	site directed mutagenesis
	5’- CGGTCTTAATA*G*CCACAATAACCAGACGACCTGCCGGATCACG -3’	
AdnA_*D935N*	5’- CGGTCTTAATAT*T*CACAATAACCAGACGACCTGCCGGATCACG -3’	
	5’- GTTATTGTG*A*ATATTAAGACCGGTAAAACCCCGGTGAGCAAAGATGATGCC -3’	
AdnB_*D1014A*	5’- CCGTAGTCG*C*TTGGAAAACCGGTAAACCGCCGCACGGTCCGG -3’	
	5’- CGGTTTTCCAA*G*CGACTACGGTCGCACCGCCGTCCGG -3’	
Cy3-labeled strand	5’- [Cyanine3]GCCCTGCTGCCGACCAACGAAGGT -3’	EMSA, Helicase assay
5’-overhang strand	5’- TTTTTTTTTTTTTTTTTTTTACCTTCGTTGGTCGGCAGCAGGGC -3’	
trap-DNA	5’- GCCCTGCTGCCGACCAACGAAGGT -3’	
ssDNA_ATPase	5’-ATTAACAATTGTTATGTCGACCACTCTGGCGATCG-3’	ATPase assay
AdnA for BamHI common	5’- attaaGGATCCATGAGCCATATTTGGGGTGTTGAAGCCGG- 3’	cloning of AdnA constructs for MPFC assay
AdnA for BstBI common	5’-attaaTTCGAAATGAGCCATATTTGGGGTGTTGAAGCCGG-3’	
common rev ClaI	5’-attaaATCGATAGCGGCCGCAAGCTTGTCGACG-3’	
common rev	5’-GCGGCCGCAAGCTTGTCGACG-3’	
motor rev ClaI	5’-attaaATCGATATCGGCCCACTGTGCAATTTCAAAG-3’	
AdnAB rev ClaI	5’-attaaATCGATCGGGGCACTGCCACGCACATG-3’	
AdnAB rev HindIII	5’-attaaAAGCTTCGGGGCACTGCCACGCACATG-3’	
Dut for MfeI	5’-attaaCAATTGttATGTCGACCACTCTGGCGATCG-3’	cloning of dut constructs for MPFC assay
Dut for EcoRI	5’-attaaGAATTCcATGTCGACCACTCTGGCGATCG-3’	
Dut rev ClaI	5’-attaaATCGATCAAACTCGCATGTCCGCCG-3’	
Dut rev HindIII	5’-attaaAAGCTTCAAACTCGCATGTCCGCCG-3’	
Int NotI for	5’-ATAAGAATGCGGCCGCTTGTAAAACGACGGCCAGTGC-3’	cloning of pLL_rec for recombination assay
Int AvrII rev	5’-ATTAACCTAGGGGTACCCGAAAGACGTCACC-3’	
KanR NotI for	5’-ATAAGAATGCGGCCGCCGCTTACAGACAAGCTGTGACC-3’	

KanR KpnI rev	5’-GGGGTACC TCAACAGGCCAGCCATTACG-3’	
pLL-rec_EcoRV for	5’-ATTAAGATATCATCGCTTGTATGGGAAGCCCC-3’	
pLL-rec EcoRV rev	5’-ATTAAGATATCCTAGATTTTAATGCGGATGTTGCGATTAC-3’	

Supplementary Table 2: Statistical analysis of differences in the derived rate constants obtained from the AdnAB nuclease assays. Pairwise comparisons were performed using two-sample t-tests.

**Difference in k value compared to AdnAB alone**	**N**	**p**	**Statistical significance at $p < 0.05$**
**FIG5**			
AdnAB	3	1	ns
AdnAB + dut	4	4.65E-04	**
**FIG6**			
AdnAB	3	1	ns
AdnAB + dut	3	0.05	*
AdnAB + dut + dUPNPP	3	0.26	ns
AdnAB + dUPNPP	2	0.13	ns
**FIG7**			
AdnAB	5	1	ns
AdnAB + dut	8	0.005	**
AdnAB+ cterm	6	0.47	ns
AdnAB+ dloop	6	0.52	ns
AdnAB + inactive	5	0.48	ns
AdnAB + BSA	3	0.15	ns
**Difference in k value compared to AdnAB+Dut**	**N**	**p**	**Statistical significance**

**FIG6**			
AdnA	3	0.05	*
AdnA + dut	3	1	ns
AdnA + dut + dUPNPP	3	0.053	ns
AdnA + dUPNPP	2	0.26	ns
**FIG7**			
AdnA	5	0.005	**
AdnA + dut	8	1	ns
AdnA + cterm	6	8.98E-04	**
AdnA + dloop	6	0.0012	**
AdnA + Inactive	5	0.002	**
AdnA + BSA	3	0.0017	**

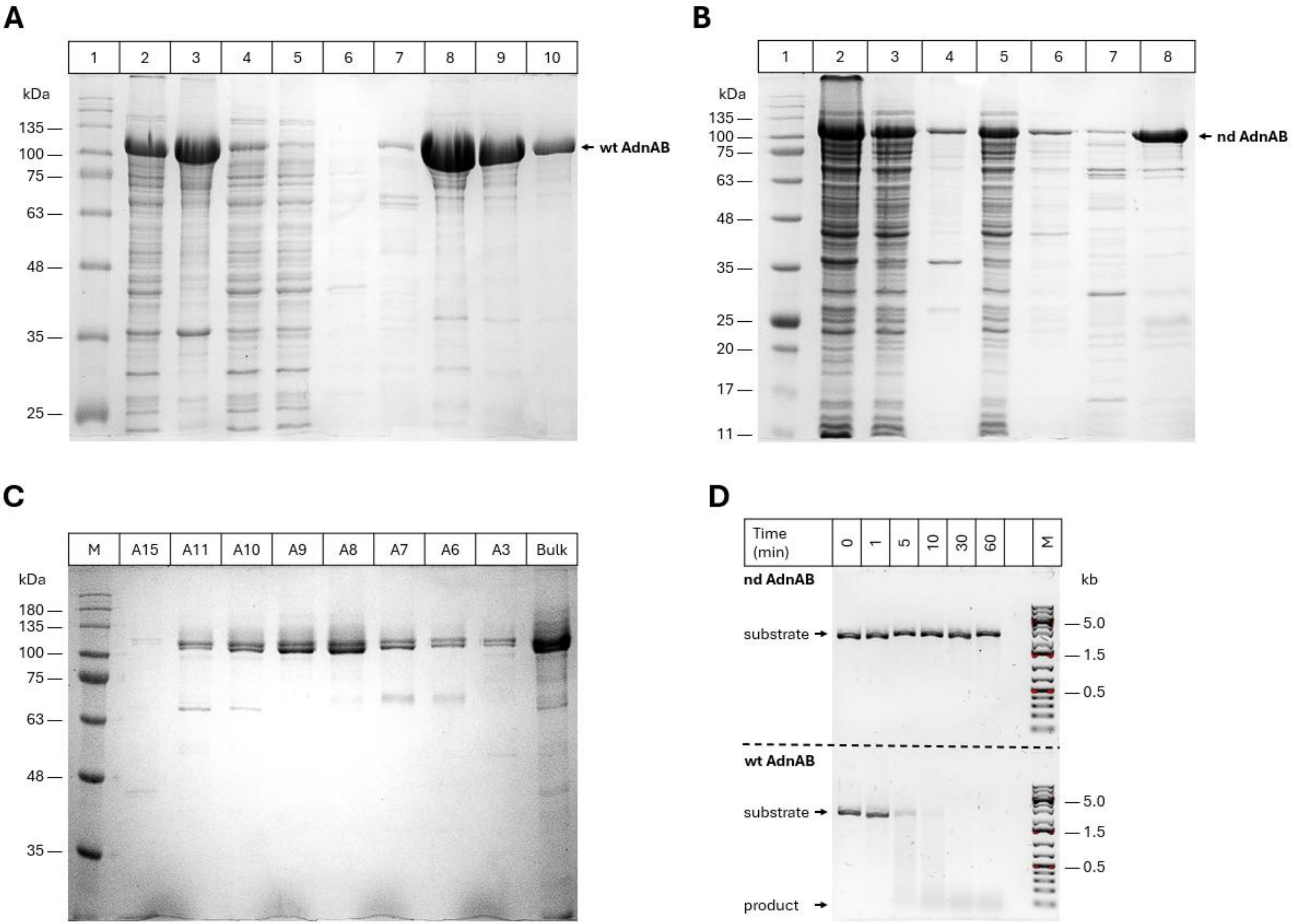
A
1
2
3
4
5
6
7
8
9
10
kDa
135
100
75
63
48
35
25
wt AdnAB
B
1
2
3
4
5
6
7
8
kDa
135
100
75
63
48
35
25
20
17
11
nd AdnAB
C
M
A15
A11
A10
A9
A8
A7
A6
A3
Bulk
kDa
180
135
100
75
63
48
35
D
Time (min)
0
1
5
10
30
60
M
nd AdnAB
kb
substrate
5.0
1.5
0.5
wt AdnAB
substrate
5.0
1.5
0.5
product

Supplementary Figure 1. Protein purification and validation of the nuclease-dead (nd) AndAB mutant

A) SDS-PAGE analysis of wt AdnAB purification. Lane 1: molecular weight marker (GRS Protein Marker MultiColour); Lane 2: IPTG-induced whole-cell lysate; Lane 3: pellet from IPTG-induced lysate; Lane 4: supernatant from IPTG-induced lysate; Lane 5: flow-through from Ni-NTA affinity column; Lane 6: wash with buffer A; Lane 7: wash with buffer A containing 40 mM imidazole; Lanes 8–10: elution fractions with buffer A containing 200 mM imidazole.

B) SDS-PAGE analysis of nd AdnAB purification. Lane 1: molecular weight marker (GRS Protein Marker MultiColour); Lane 2: IPTG-induced whole-cell lysate; Lane 3: supernatant of IPTG-induced cell lysate; Lane 4: pellet of IPTG-induced cell lysate; Lane 5: flow-through of the Ni-NTA affinity column loading; Lane 6: wash with buffer A; Lane 7: wash with buffer A containing 40 mM imidazole; Lane 8: elution of nd AdnAB in buffer A containing 200 mM imidazole.

C) SDS-PAGE analysis of size-exclusion chromatography fractions of TEV-cleaved nd AdnAB. Bulk: sample loaded onto the column; Fractions A3–A15: decreasing molecular weight complexes, with A8–A9 corresponding to the expected 230 kDa heterodimer. These fractions were used for EMSA and helicase assays.

D) Nuclease activity assay of nd AdnAB compared to wt AdnAB. The nd AdnAB does not cleave the DNA substrate, whereas wt AdnAB digests most of the substrate within 5 min. Samples were collected at the indicated time points and analysed by gel electrophoresis in a 1% agarose gel. "M" designates the Thermo Scientific GeneRuler 1 kb Plus DNA Ladder.

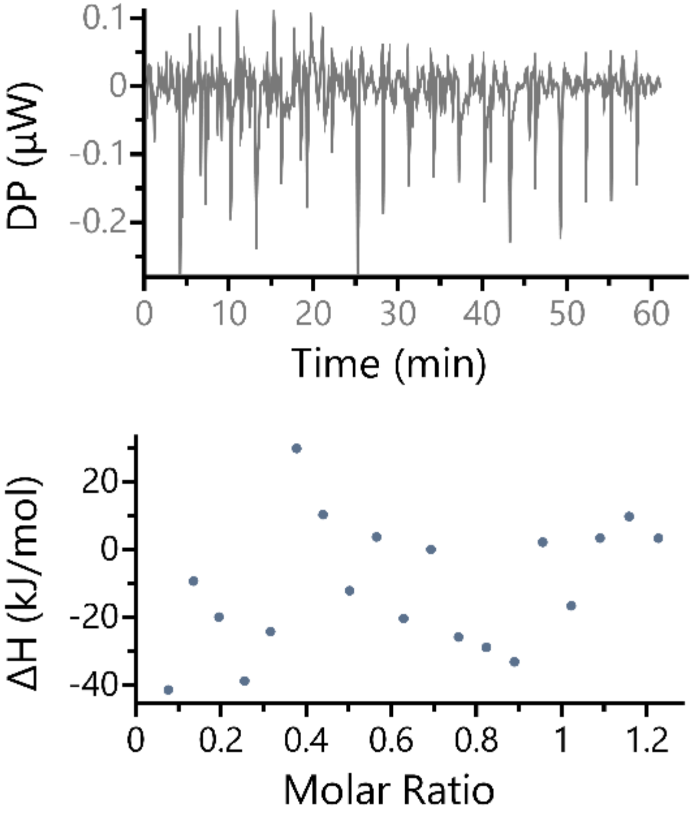

Supplementary Figure 2. ITC measurement assessing the potential interaction of AdnA and Dut in the absence of DNA. Heat changes were recorded upon titration of 42.8 µM Dut (monomer concentration) into 3.9 µM AdnAB in buffer A. The thermogram shows no consistent binding trend, indicating the absence of detectable interaction between AdnAB and Dut under these conditions.

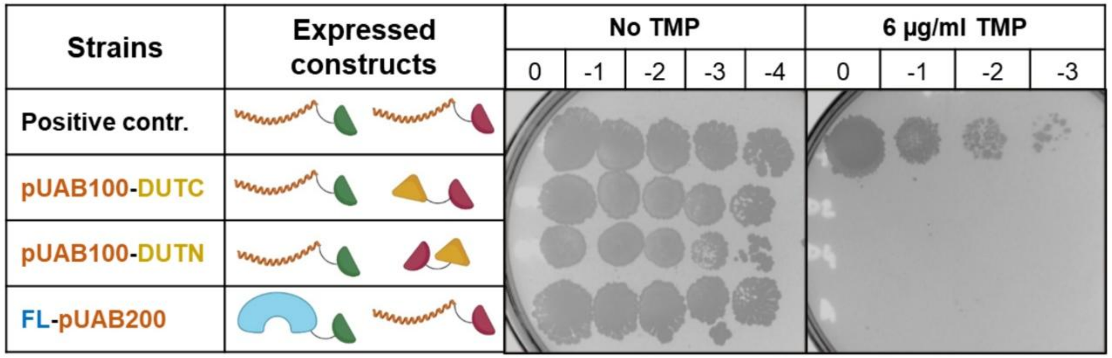

Supplementary Figure 3. MPFC assay controls

Viability of the different control strains in non-selecting and selecting media containing Tmp. Cells were applied in different dilutions. 0 denotes the undiluted culture; -1, -2, -3 and -4 denote 10, 100, 1000 and 10000x dilutions, respectively. The experiment was repeated three times. FL, full-length AndA; DUTC, dUTPase fused to DHFR at its C-terminus; DUTN, dUTPase fused to DHFR at its N-terminus. The illustration has been produced using Biorender.

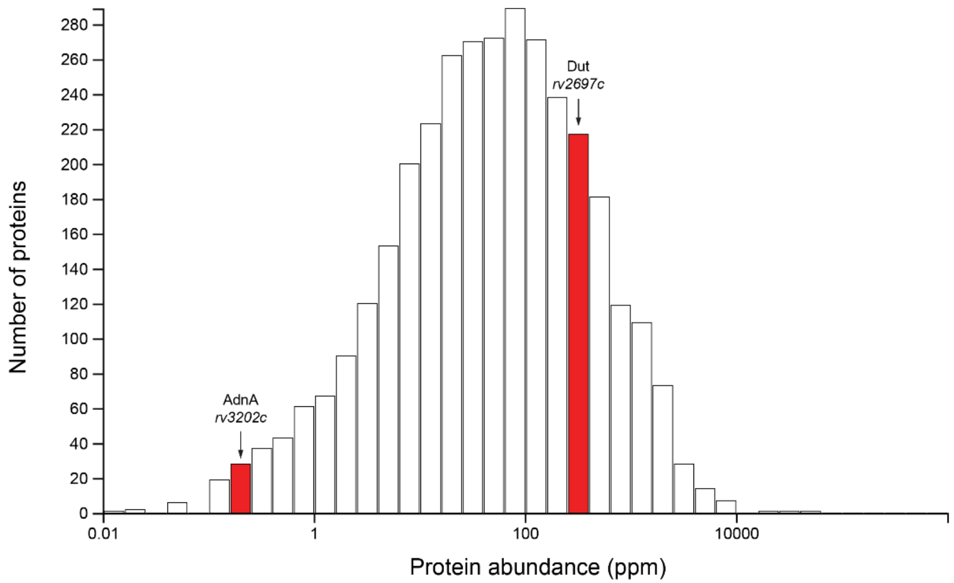

Supplementary Figure 4. Relative abundance of Dut and AdnA in *Mycobacterium tuberculosis* H37Rv. Protein abundance values were obtained from the PaxDb Protein Abundance Database. AdnA (Rv3202c) is expressed at very low levels (0.21–0.79 ppm), ranking in the lowest 5% of quantified proteins, whereas Dut (Rv2697c) shows markedly higher abundance (274–433 ppm), placing it in the top quartile of the proteome. Source: Dut: https://pax-db.org/protein/83332/Rv2697c; AdnA: https://pax-db.org/protein/83332/Rv3202c
